# PVP Passivated δ-CsPbI_3_: Vacancy Induced Visible-Light Absorption and Efficient Photocatalysis

**DOI:** 10.3390/molecules29071670

**Published:** 2024-04-08

**Authors:** Jianfeng Wen, Xin Du, Feng Hua, Yiting Gu, Ming Li, Tao Tang

**Affiliations:** 1 College of Physics and Electronic Information Engineering, Key Laboratory of Low-Dimensional Structural Physics and Application, Education Department of Guangxi Zhuang Autonomous Region, Guilin University of Technology, Guilin 541004, China; wjfculater@163.com (J.W.); duxin0727@126.com (X.D.); huafeng6026@163.com (F.H.); yiting10596@163.com (Y.G.); liming928@163.com (M.L.); 2 School of Electronic Information and Automation, Guilin University of Aerospace Technology, Guilin 541004, China

**Keywords:** perovskite, δ-CsPbI_3_, surface passivation, photodegradation

## Abstract

The aqueous instability of halide perovskite seriously hinders its direct application in water as a potential photocatalyst. Here, we prepared a new type of polyvinylpyrrolidone (PVP) passivated δ-CsPbI_3_ (δ-CsPbI_3_@PVP) microcrystal by a facile method. This material can be uniformly dispersed in water and stably maintain its crystal structure for a long time, breaking through the bottleneck of halide perovskite photocatalysis in water. Under visible light, δ-CsPbI_3_@PVP can almost completely photodegrade organic dyes (including Rhodamine B, methylene blue, and crystal violet) in only 20 min. The efficient photocatalytic activity is attributed to the enhanced visible light absorption arising from PbI_2_ defects in δ-CsPbI_3_@PVP and the intrinsic low photoluminescence quantum yield of δ-CsPbI_3_, which induces efficient light absorption and photocatalytic activity. We highlight δ-CsPbI_3_@PVP as an effective aqueous photocatalyst, and this study provides new insights into how to exploit the potential of halide perovskite in photocatalytic applications.

## 1. Introduction

In recent decades, semiconductor-based photocatalysis has attracted much attention, including metal oxides [1,2], metal sulfides [3,4,5], and two-dimensional layered materials [6,7,8,9]. Among metal oxides, TiO_2_ [10] and ZnO [11] have become the most popular photocatalysts due to their low cost, high activity, and stable chemical properties. However, they possess wide band gaps of 3.2–3.4 eV, which can only absorb UV light, accounting for merely 4% of the sunlight that can be utilized. Among metal sulfides, CdS [12] and ZnS [5] are the most studied. CdS has a band gap of 2.4 eV and can capture visible light, but it is limited by a strong photo corrosion effect. ZnS is inert in corrosive environments, but its band gap of 3.6 eV again leads to poor visible light absorption. Two-dimensional layered materials such as g-C_3_N_4_ and MXenes are prospective photocatalysts due to their large specific surface area. However, g-C_3_N_4_ fails to cover most visible light [13,14] and MXenes possess low photocatalytic activity [15]. Moreover, all these traditional photocatalysts suffer from the low separation and transfer efficiency of the photogenerated electron–hole pairs. Therefore, in the past two decades, researchers have devoted much effort, including in morphology and size control [16,17], element doping [18], and material compositing [19], to facilitate the separation and transfer of the photogenerated carriers. Nevertheless, the current visible light photocatalytic performances of these materials are still far away from expectations.

As a rising star material, halide perovskite has been widely studied in solar cells [20], light-emitting diodes (LEDs) [21], lasers [22], and photodetectors [23,24] due to its simple synthesis, wide light absorption range, high extinction coefficient, long carrier life and transmission distance, and adjustable band structure [25]. Also, halide perovskite has been successfully used in carbon dioxide reduction [26,27], hydrogen evolution [28,29,30,31,32,33], and organic dye photodegradation [34,35,36,37,38]. However, due to the aqueous instability of halide perovskite, such photocatalytic experiments have to be conducted in organic solvents [35,38,39,40,41,42,43,44,45,46,47,48,49] or after water-resistant-surface passivation [28,29,30,31,32,33,50,51]. Searching for halide perovskite, which can be directly used in water, is important in photocatalysis. In the perovskite family, δ-phase perovskite is well known for its stability in humidity and unsuitability for optoelectronic applications arising from its poor photoluminescence (PL) quantum yield (QY) [52]. Interestingly, the band gap of δ-CsPbI_3_ is 2.54 eV [53], combined with a high light absorption coefficient and low PLQY, implying that its photo-generated charge carriers are less likely to undergo direct electron–hole recombination and are more likely to effectively participate in photocatalytic reactions. These unique characteristics suggest that δ-CsPbI_3_ may be a promising photocatalyst.

Here, via a facile method, we synthesized δ-CsPbI_3_ microcrystals and passivated them by polyvinylpyrrolidone (PVP) to enhance their dispersibility and stability in water. Such δ-CsPbI_3_@PVP can stably exist in water for at least 30 days without obvious deterioration. It exhibited a striking visible light photodegradation ability for organic dyes, namely, completely photodegrading Rhodamine B (RhB), methylene blue (MB), and crystal violet (CV) in only 20 min. Such a high efficiency was highly related to the enhanced absorption in the visible region induced by the PbI_2_ vacancy defects in δ-CsPbI_3_@PVP and its intrinsic low PLQY, since adequate photocarriers can be generated and participate in the photocatalytic process to effectively degrade organic dyes. The δ-CsPbI_3_@PVP material showed good stability in cyclic tests, indicating its resistance to light corrosion. Our results opened a gate on how to develop the photocatalysis of halide perovskites.

## 2. Results

**Figure 1:**
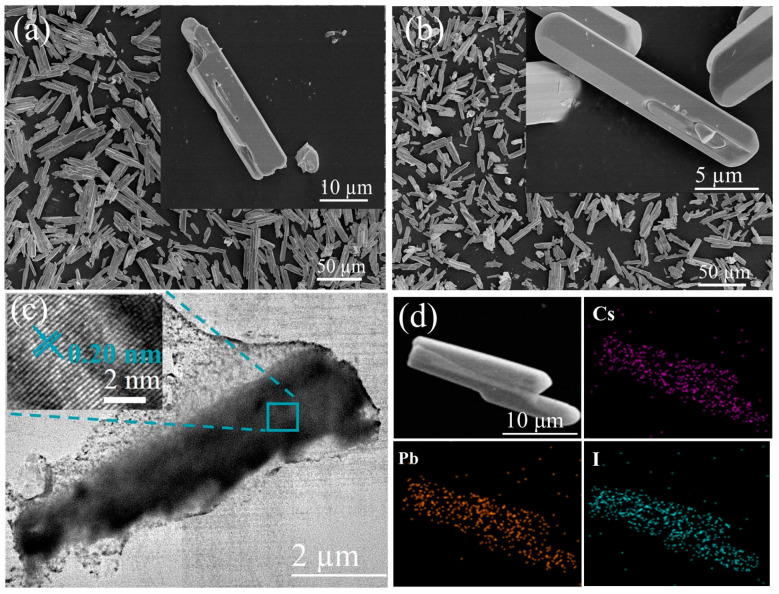
SEM images of (**a**) δ-CsPbI_3_ and (**b**) δ-CsPbI_3_@PVP microcrystals. The insets are the corresponding high-resolution images. (**c**) Typical TEM image of a δ-CsPbI_3_@PVP microcrystal. The inset is the corresponding high-resolution TEM (HRTEM) image. (**d**) EDS elemental mapping of δ-CsPbI_3_@PVP microcrystals.

Shown in Figure 1a,b are the SEM images of δ-CsPbI_3_ and δ-CsPbI_3_@PVP microcrystals. They both are demonstrated as long stripes, with an average length of 22.5 (Figure S1a, δ-CsPbI_3_) and 20 microns (Figure S1b, δ-CsPbI_3_@PVP), respectively. Figure 1c shows a typical TEM image of δ-CsPbI_3_@PVP. It can be clearly seen that, the long-stripe-shaped δ-CsPbI_3_ was covered by the adherent PVP, which thus enhanced the dispersion and stability of δ-CsPbI_3_ in water. The inset HRTEM image of δ-CsPbI_3_@PVP shows a lattice spacing of 0.20 nm, which corresponds to the (414) crystal faces of δ-CsPbI_3_ microcrystals. The elemental analyses (Figure 1d, δ-CsPbI_3_@PVP) showed the atomic ratio of Cs/Pb/I = 1:0.65:2.45, basically according with the formula of halide perovskites with some defects.

**Figure 2:**
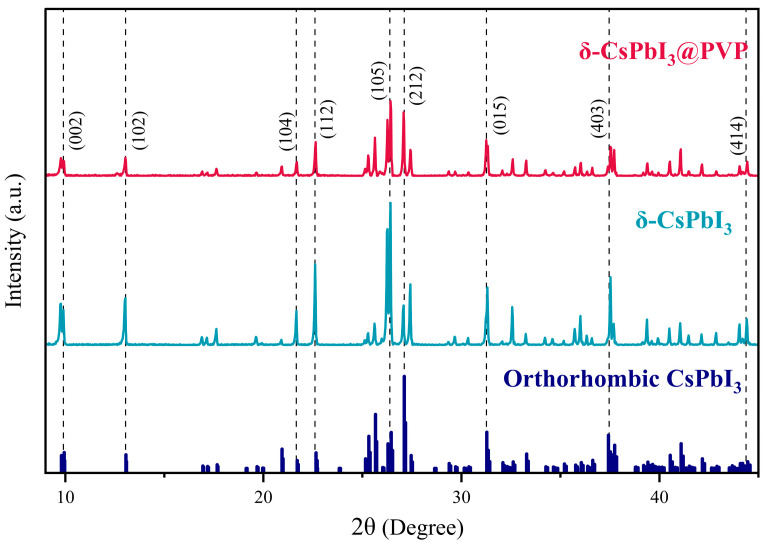
XRD patterns of δ-CsPbI_3_ and δ-CsPbI_3_@PVP microcrystals.

Figure 2 shows the XRD patterns of δ-CsPbI_3_ and δ-CsPbI_3_@PVP microcrystals. One can see that δ-CsPbI_3_ and δ-CsPbI_3_@PVP are almost the same, demonstrated as an orthorhombic structure (Pnma space group) and well-matched with the standard PDF card, as found in other studies of the δ-phase CsPbI_3_[54]. The results indicated that the PVP passivation did not change the crystal structure of δ-CsPbI_3_. Although a previous study showed that the amide group in PVP may reduce the surface energy to impose an effect on the crystal structure of CsPbI_3_ [55], here we still deem δ-CsPbI_3_@PVP as PVP-wrapped orthorhombic δ-phase CsPbI_3_ crystals. Elemental analyses by EDS of the δ-CsPbI_3_@PVP crystals showed that the atomic ratio of Cs/Pb/I = 1:0.65:2.45 (Figure 1d), which basically was in accordance with the molecular formula of halide perovskites, however with some defects present.

**Figure 3:**
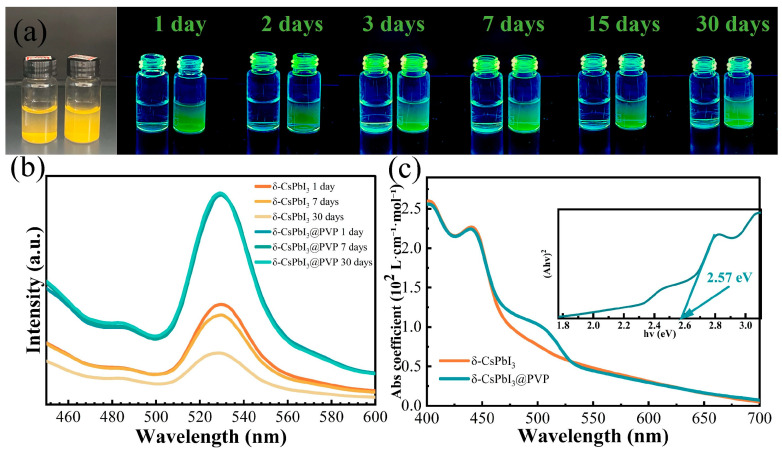
(**a**) Optical images under sun and UV light of δ-CsPbI_3_ (left) and δ-CsPbI_3_@PVP (right) water solutions. (**b**) Time-dependent PL spectra of δ-CsPbI_3_ and δ-CsPbI_3_@PVP aqueous solutions. The excitation wavelength is 390 nm. (**c**) Absorption coefficients of δ-CsPbI_3_ and δ-CsPbI_3_@PVP. The inset is the Tauc plot of δ-CsPbI_3_@PVP.

The aqueous dispersion and stability of photocatalysts are the important factors determining whether they are suitable for photodegrading organic dyes in water. In Figure 3a, it is found that only the newly synthesized δ-CsPbI_3_ microcrystals could be dispersed in water, and they soon tended to aggregate, so using δ-CsPbI_3_ microcrystals as water-based photocatalysts was challenging. In contrast, the dispersion of δ-CsPbI_3_@PVP microcrystals was pretty good without notable aggregation. In Figure 3b, after 30 days, the photoluminescence (PL) spectrum shape and intensity of δ-CsPbI_3_@PVP barely changed, demonstrating the fantastic water stability of CsPbI_3_@PVP microcrystals. Obviously, the PVP passivation of perovskite surface improved the dispersion and stability of perovskite in water [36]. The PL center of the δ-CsPbI_3_@PVP water solution was 530 nm, demonstrating the typical green light due to self-trapped excitons of δ-phase CsPbI_3_ microcrystals [56]. Its UV-vis absorption spectrum [Figure 3c] also showed typical δ-CsPbI_3_ behavior, with a broad absorption range in the visible region and a clear band-to-band absorption edge close to 460 nm (2.57 eV), which was consistent with other studies on δ-CsPbI_3_ [56,57]. Notably, δ-CsPbI_3_@PVP had a significant absorption shoulder close to 530 nm. We also performed PL measurements on δ-CsPbI_3_@PVP powder (Figure S2). The δ-CsPbI_3_@PVP powder demonstrated a broad emission spectrum ranging from 437 to 660 nm, which was in accordance with the previous literature [53,58]. Even so, two peaks also could be clearly distinguished, which were located at about 460 nm and 530 nm, respectively. Similar to its solution counterpart, the former peak was ascribed to band-to-band transition, and the latter was due to the self-trapped excitonic emission. Obviously, the band-to-band emission of δ-CsPbI_3_@PVP was totally suppressed in water; however, its specific reason remains unclear, and we speculate it is highly related to the aqueous environment. Since the scope of this work is studying the aqueous photocatalysis of δ-CsPbI_3_, the following discussion is mainly based on the optical properties of the sample water solution.

**Figure 4:**
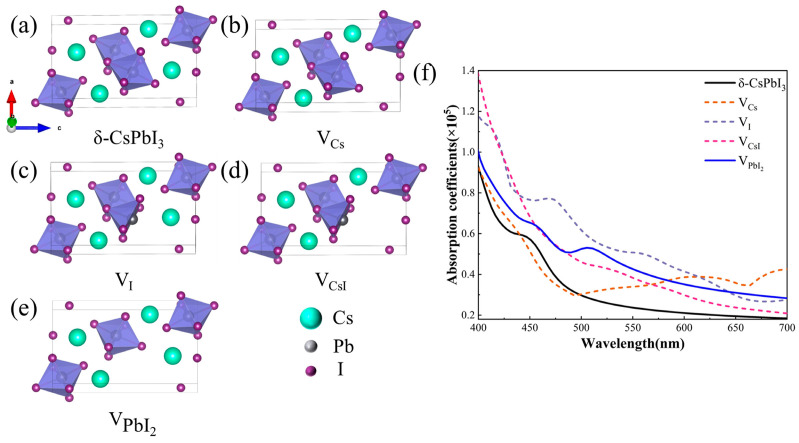
(**a**) δ-CsPbI_3_ cell model, (**b**) Cs vacancy cell model, (**c**) I vacancy cell model, (**d**) CsI vacancy cell model, (**e**) PbI_2_ vacancy cell model, (**f**) calculated absorption coefficient maps for δ-CsPbI_3_ and δ-CsPbI_3_ at four vacancies, V_Cs_, V_I_, V_CsI_, and $V_{{\mathrm{PbI}}_{2}}$.

As known, δ-CsPbI_3_ is generally the deteriorated product of its perovskite-phase counterpart when exposed to moisture. Water can dissolve some ions and produce vacancy defects in perovskite and eventually result in the δ-phase. Hence, δ-CsPbI_3_ should be a material rich in vacancies, at least on its crystal surface. The absorption shoulder at ~530 nm (Figure 3) should be highly related to such defects. Therefore, we calculated the changes in optical properties when four common vacancy defects (V_Cs_, V_I_, V_CsI_, and $V_{{\mathrm{PbI}}_{2}}$) were present (Figure 4) [58]. Figure 4a–e respectively depict the structures of pristine and defective δ-CsPbI_3_ cells. Figure 4f shows that such vacancy defects can all result in the enhancement of the absorption coefficient in the visible range (460–600 nm). Notably, $V_{{\mathrm{PbI}}_{2}}$ brings about a prominent absorption shoulder close to 530 nm, similar to our experimental results [Figure 3c]. Combined with the EDS results in Figure 1d, reasonably, we speculate that δ-CsPbI_3_@PVP crystals contain many vacancy defects, primarily dominated by PbI_2_ vacancies. It is also noted in Figure 3c that δ-CsPbI_3_ without a PVP coating showed no prominent absorption shoulder at ~530 nm in water, which may have been due to the further water corrosion of vacancy defects in δ-CsPbI_3_. Such PbI_2_ vacancy defects were protected by PVP in δ-CsPbI_3_@PVP; thus, the absorption characteristics at ~530 nm could be exhibited.

The absorption coefficients of the δ-CsPbI_3_@PVP water solution were larger than 2 × 10^2^, 0.5 × 10^2^, and 0.2 × 10^2^ L•mol^−1^•cm^−1^ in the visible range of 400–460, 460–530, and 530–700 nm, respectively [Figure 3c]. We compared it with two famous photocatalysts, g-C_3_N_4_ and TiO_2_; although, these two are more reasonably deemed suspended rather than soluble in water. As shown in Figure S3, in the visible light region, g-C_3_N_4_ had only a band-to-band absorption coefficient of ~0.5 × 10^2^ L•mol^−1^•cm^−1^ in the wavelength region of 400–420 nm. When the wavelength exceeded 430 nm, its absorption coefficient soon dropped to less than 0.1 × 10^2^ L•mol^−1^•cm^−1^. The star photocatalyst in the UV region was TiO_2_, of which the absorption coefficient was less than 0.5 × 10^2^ L•mol^−1^•cm^−1^, even in the UV region (Figure S3). Undoubtedly, the strong absorption ability of δ-CsPbI_3_@PVP is important to its visible light photocatalysis. As known, most members in the halide perovskite family have a very high PLQY; that means most photogenerated carriers will directly recombine to emit light, which is obviously not conducive to photocatalytic activity. Fortunately, δ-phase perovskite is notorious for its weak PL intensity [56], which herein would be a merit since plentiful indirectly recombined photocarriers could participate in the photodegradation process. Using quinine sulfate as a reference, we found that the PLQYs of δ-CsPbI_3_ and δ-CsPbI_3_@PVP were only 0.17% and 0.43% (see Table 1). Its strong absorption, low PLQY, good dispersion, and stability in water indicated that δ-CsPbI_3_@PVP has great potential in the photocatalytic field.

**Figure 5:**
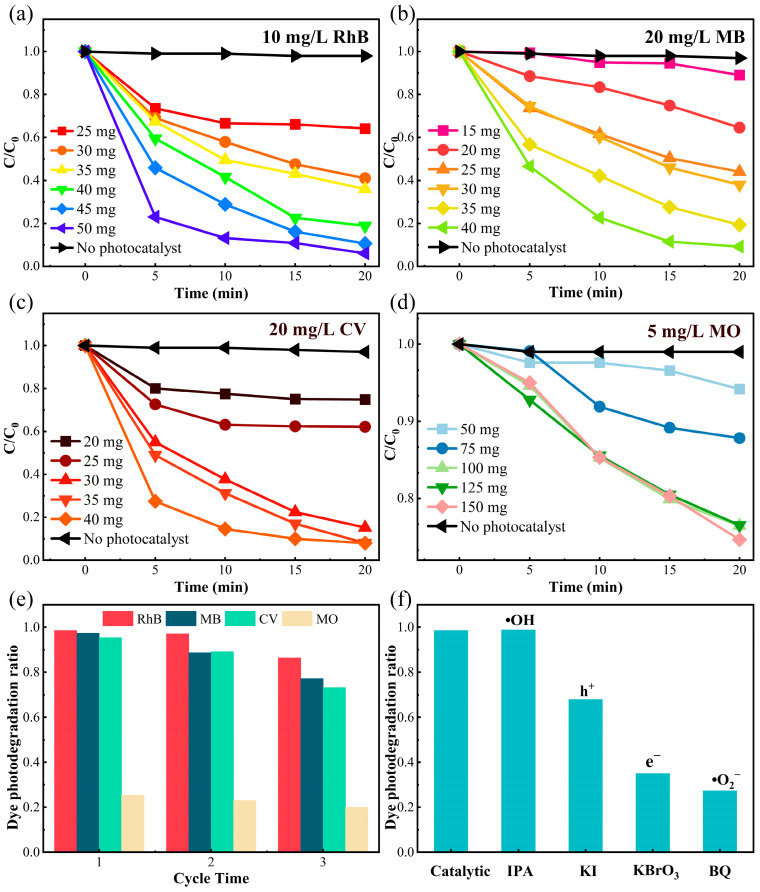
Visible light catalytic degradation of δ-CsPbI_3_@PVP on different organic dyes (50 mL). The concentration variations of (**a**) 10 mg/L RhB, (**b**) 20 mg/L MB, (**c**) 20 mg/L CV, and (**d**) 5 mg/L MO under photodegradation with different δ-CsPbI_3_@PVP qualities. C and C_0_ refer to the current and initial concentration of dyes, respectively. (**e**) Cyclic photodegradation experiments on different dyes. (**f**) Effects of different free radical quenchers.

Based on the above conjecture, we conducted visible light photodegradation experiments on δ-CsPbI_3_@PVP microcrystals’ effects on different organic dyes in water. Four representative dyes, 10 mg/L RhB, 20 mg/L MB, 20 mg/L CV, and 5 mg/L methyl orange (MO), were used as degradation references after 30 min in the dark to achieve an equilibrium of absorption–desorption. As shown in Figure 5a–d, small amounts of δ-CsPbI_3_@PVP (≤50 mg) could photodegrade 99% of RhB, MB, and CV in merely 20 min, but only 25% of MO could be degraded even after increasing the quality of δ-CsPbI_3_@PVP to 150 mg. The exact reason why δ-CsPbI_3_@PVP barely photodegraded MO is hard to know; therefore, this thesis focused on the photodegradation of RhB, MB, and CV by δ-CsPbI3@PVP. As expected, the photodegradation ability increased with increasing the mass of the photocatalyst. To avoid any possible deviations on the concentration calibration via absorption spectroscopic measurements of organic dyes, we performed total organic carbon (TOC) experiments and found that all the mineralization of RhB, MB, and CV could exceed 90% after 20 min of photodegradation. After three cycles [Figure 5e], the degradation capacity of δ-CsPbI3@PVP remained stable, and the small decline was attributed to the photo-corrosion of the photocatalyst caused by the prolonged reaction. It was noted that the photo-degradation ability of δ-CsPbI_3_@PVP was insusceptible to the PH values of the solution; in the PH range of 3–11, δ-CsPbI_3_@PVP all could degrade 99% of RhB in 20 min (Figure S4).

In order to explore its catalytic process, free radical trapping experiments were carried out. Different trapping agents were added to find the led active species in the catalytic process. P-benzoquinone (BQ), isopropyl alcohol (IPA), potassium iodide (KI), and potassium bromate (KBrO_3_) were respectively used to scavenge superoxide free radicals (•O_2_^−^), hydroxyl free radicals (•OH), holes (h^+^), and electrons (e^−^). As shown in Figure 4f, the photocatalytic performance was significantly reduced after the addition of BQ (71%) and KBrO_3_ (63%), indicating that •O_2_^−^ and e^−^ were the predominant active species in this photocatalytic process. After the holes were captured, the photodegradation efficiency also had a 30% decrease. Adding IPA had no influence on the photodegradation, which meant that in this process, •OH radicals were hardly produced. 

In addition, Table 2 compares the photocatalytic activity of δ-CsPbI_3_@PVP with that of other perovskite materials. Previously, it has been reported in other studies that to maintain the stability of perovskites, photocatalytic experiments must be conducted solely in organic solvents. In contrast, the δ-CsPbI_3_@PVP material was capable of direct utilization in water. Furthermore, under visible light, δ-CsPbI_3_@PVP effectively degraded various organic dyes (such as RhB, MB, and CV) within a mere 20 min timeframe. It can be seen that the photocatalytic activity of the δ-CsPbI_3_@PVP material was far ahead of those of other reported studies.

## 3. Discussion

A good photocatalyst is preferred to have a good light absorption ability and good redox ability [59]. A good absorption ability generally requires a narrow band gap, while a good redox ability favors a high conduction and/or low valence band level, which is convenient for the transfer of photogenerated electrons and/or holes, namely, a wide band gap [59]. Obviously, there is a contradiction between these; however, in δ-CsPbI_3_@PVP, a good compromise was achieved. Its band gap was 2.57 eV [the inset of Figure 3c], which was a wide band gap among the visible light semiconductor photocatalysts. Meanwhile, it also had a strong light absorption ability no matter above or below the band gap [Figure 3c]. Especially, the existence of self-trapping states further enhances the absorption below the band gap. Large amounts of photons were absorbed and turned into transferable, indirectly recombined electrons and holes (PLQY is 0.43%, Table 1), together with its good dispersion and stability in water, which make δ-CsPbI_3_@PVP become a highly efficient visible light photocatalyst.

**Figure 6:**
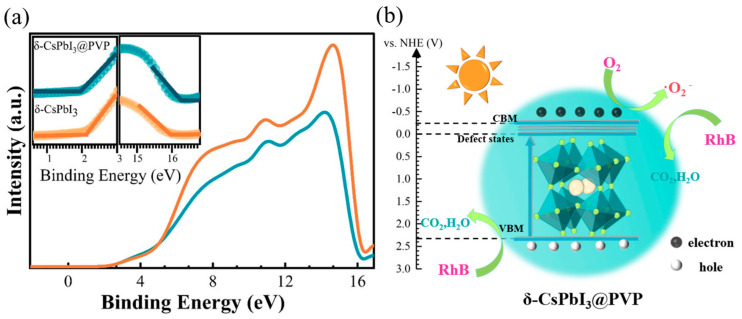
(**a**) UPS spectra of δ-CsPbI_3_ and δ-CsPbI_3_@PVP microcrystals. The insets are the corresponding onset and cutoff parts. (**b**) RhB degradation process of δ-CsPbI_3_@PVP microcrystals under visible light.

Shown in Figure 6 is the schematic diagram of the RhB photodegradation process of δ-CsPbI_3_@PVP. Using a vacuum level as a reference, the UPS measurements [Figure 6a] could define the valence band maximum (VBM) by E_VBM_ = −[21.22 − (E_cutoff_ − E_onset_)]. For δ-CsPbI_3_@PVP, the E_cutoff_ was 16.30 eV and the E_onset_ was 1.90 eV, so the E_VBM_ was −6.82 eV. Considering the band gap was 2.57 eV, one can know that the conduction band maximum (CBM) was −4.25 eV. The emissive center was located at 530 nm, implying the energy difference between the trap states and VBM was 2.34 eV, namely, the energy level of trap states was −4.48 eV. Conventionally, we expressed the energy levels using a normal hydrogen electrode (NHE) as a reference [E(NHE) = −4.5 − E(vacuum), which represented the relationship between the NHE and vacuum energy levels], and the energy levels of the CBM, trap states, and VBM were −0.25, −0.02, and 2.32 eV, respectively. The photocatalytic mechanism of δ-CsPbI_3_@PVP could be reasonably inferred according to the experimental results:

(1) $${\delta -\mathrm{CsPbI}}_{3}@\mathrm{PVP}+\mathrm{dyes}\;\to \;*\mathrm{dyes}$$

(2) $${\delta -\mathrm{CsPbI}}_{3}@\mathrm{PVP}+h\nu \;\to h^{+}+e^{-}$$

(3) $$O_{2}+e^{-}\to \;•O_{2^{-}}$$

(4) $$•O_{2^{-}}+*\mathrm{dyes}\to \;\mathrm{mineralization}\;\mathrm{products}$$

where *dye denotes the dye molecule activated by δ-CsPbI_3_@PVP. When δ-CsPbI_3_@PVP was exposed to visible light, the absorption of photons caused the electrons to be excited from the VB to the CB and trap states, resulting in photogenerated electrons and holes. The electrons were captured by O_2_ to produce •O_2_^−^, and then •O_2_^−^ reacted with the *dye to produce mineralized compounds. If the electrons or •O_2_^−^ radicals were captured, the photodegradation efficiency would be significantly decreased [Figure 5f]. Moreover, we could not completely neglect the effects of photogenerated holes [Figure 5f] since they also can directly oxidize and degrade dyes [60]. 

## 4. Experimental Section

### 4.1. Materials

CsI (99.9%), PbI_2_ (99%), polyvinylpyrrolidone (PVP, K29-32), MB, CV, and methyl orange (MO) were purchased from Roan (Olds, AB, Canada). RhB was purchased from the Tianjin Guangfu fine chemical research institute (Tianjin, China). Ethanol (>99.7%, AR) and isopropyl alcohol (IPA, 99.7%, AR) were purchased from Silon Science (Allentown, PA, USA). Potassium iodide (KI, ≥99.0%, AR), potassium bromates (KBrO_3_, ≥99.8%, AR), P-benzoquinone (BQ, 99%), and hydroiodic acid (HI, 55.5–58.0% with <1.5% H_3_PO_2_ stabilizer) were from Aladdin (Shanghai, China). The water was deionized.

### 4.2. Synthesis of δ-CsPbI_3_ and δ-CsPbI_3_@PVP

PbI_2_ (10 mmol, 4.61 g) was dissolved in hydroiodic acid (8 mL), and then CsI (10 mmol, 2.59 g, dissolved in 3 mL of water) was added dropwise to produce a brown precipitate. The precipitate was filtered, washed twice with ethanol, and dried in a vacuum oven at 60 °C for 12 h. Then, the δ-CsPbI_3_ microcrystals were obtained.

Adding the above-mentioned brown precipitate with 2 g PVP to 30 mL of ethanol, a suspension was formed after vigorous stirring at room temperature for 30 min. After 15 min of ultrasonication, the precipitation was filtered and then washed with ethanol and water, finally dried in a vacuum oven at 60 °C for 12 h to obtain δ-CsPbI_3_@PVP microcrystals.

### 4.3. Characterization

The morphologies of δ-CsPbI_3_ and δ-CsPbI_3_@PVP samples were characterized by a transmission electron microscope (TEM, JEM-2100F, JEOL, Akishima, Japan) and a field emission scanning electron microscope (SEM, S-4800, Hitachi, Tokyo, Japan) equipped with an energy dispersive spectrometer (EDS). The crystal structures of δ-CsPbI_3_ and δ-CsPbI_3_@PVP were determined by X-ray diffraction (XRD, X ‘Pert PRO, JEOL, Tokyo, Japan). UV photoelectron spectroscopy (UPS) measurements were performed on a photoelectron spectrometer (ESCALAB 250Xi, Thermo Scientific, Waltham, MA, USA) with a He I source of 21.22 eV. A UV spectrophotometer (UV2700, Shimadzu, Kyoto, Japan) and fluorescence spectrometer (Edinburgh FL/FS900 Carry Eclipse, Agilent, Santa Clara, CA, USA) were used to determine the UV-vis absorption spectra, photoluminescence (PL) spectra, and relative quantum yield (QY). 

Under the same excitation, the fluorescence spectra of a quinine sulfate solution and sample were tested, and the PLQY was calculated according to the following formula:

(5) $$\Phi_{x}=\frac{n_{x}^{2}}{n_{QS}^{2}}\cdot \frac{A_{QS}\cdot I_{x}}{A_{x}\cdot I_{QS}}\cdot \Phi_{QS}$$

where $\Phi_{QS}$ is the fluorescence quantum yield of the standard reference quinine sulfate solution (54%). *A_QS_*, *I_QS_*, *n_QS_*, and *A_x_*, *I_x_*, *n_x_* refer to the absorbance value, integral area, and solution refractive index of the quinine sulfate solution and the sample, respectively.

We used the molar absorption coefficient (*ε*, L•mol^−1^•cm^−1^) to compare the absorption abilities of different solutions. The absorbance (*A*) was determined by a UV-vis spectrophotometer, and the *ε* was calculated as follows:

(6) $$\varepsilon =\frac{A}{bc}$$

Among these, *c* is the concentration (mol/L), and the optical path *b* was 1 cm. In this work, the concentrations of δ-CsPbI_3_ and δ-CsPbI_3_@PVP were both$\;4.16\times 10^{-3}\;\mathrm{mol}/L$.

### 4.4. Photocatalytic Activity Measurements 

The photocatalytic activity of the δ-CsPbI_3_@PVP samples was studied by testing the degradation of RhB, MB, MO, and CV dyes under visible light. A xenon lamp (PLS-SXE300, 300 W, λ > 420 nm) was the visible light source. A certain quality of the δ-CsPbI_3_@PVP sample (15–150 mg) was placed in 50 mL of dye aqueous solution (5–20 mg/L); then, the mixed solution was stirred in the dark for 30 min to achieve an adsorption–desorption equilibrium. Thereafter, every 5 min, the 3 mL solution was taken out to determine the dye concentration by a UV-visible spectrophotometer (UV2700, Shimadzu). The photocatalysts were repeatedly collected by centrifugation and drying and then used for the recycle photodegradation experiments. A total organic carbon (TOC) analyzer (Shimadzu, TOC-L CPN) was also used to determine the photodegradation efficiency of the organic dyes.

The concentration of the organic dyes was determined by a UV-vis spectrophotometer, and the degradation efficiency of the organic dyes was calculated as follows:

(7) $$\mathrm{degradation}\;\mathrm{ratio}\;(\%)=\frac{C_{0}-\;C}{C_{0}}\times 100=\frac{A_{0}-\;A}{A_{0}}\times 100$$

where *C*_0_ and *C* are the initial and real-time concentrations of the organic dyes, respectively, and *A*_0_ and *A* are the initial and real-time absorbance of RhB at 554 nm (MB at 660 nm, MO at 463 nm, and CV at 583 nm), respectively.

### 4.5. Computational Details 

The calculations in this paper were based on the Vienna Ab initio Simulation Package (VASP), calculated by Density Functional Theory (DFT) [59]. Generalized gradient approximation (GGA) and Perdew–Burke–Ernzerhof (PBE) were used to describe the correlation terms of the computational model. Electron–ion interactions were described using the projection-enhanced wave potential (PAW) [60]. A correction for van der Waals force interactions in the crystal cell model using Grimme’s DFT-D3 approach [61] was performed. The structure of δ-CsPbI_3_ was orthorhombic, and its lattice constants were a = 10.46 Å, b = 4.80 Å, and c = 17.78 Å, respectively. In this paper, a 1 × 1 × 1 cell model was established, in which there were 20 atoms, including 4 Cs, 4 Pb, and 12 I atoms. The plane-wave cut-off energy was set to 450 eV. An amount of 2 × 2 × 1 K points in the Brillouin zone were sampled using the Gamma method, and non-self-consistent calculations were performed using a 4 × 4 × 1 grid. Then, on the basic structure of the intrinsic perovskite model, the absorption coefficient variation caused by common vacancy defects were calculated. V_Cs_, V_I_, V_CsI_, and $V_{{\mathrm{PbI}}_{2}}$ referred to the vacancy induced by the removal of a single Cs atom, a single I atom, a Cs atom and an I atom, and a Pb atom and two I atoms in the cell. These vacancy defects are commonly generated during the preparation of halide perovskites. During the optimization process, the convergence energy and force per atom were 10^−5^ eV and 0.05 eVÅ^−1^, respectively, and all the atoms of the system (including atomic positions and lattice constants) remained fully relaxed.

## 5. Conclusions

In summary, we successfully synthesized water-stable δ-CsPbI_3_@PVP microcrystals to photodegrade various organic dyes in aqueous photocatalytic systems. These δ-CsPbI_3_@PVP microcrystals exhibited a long stripe shape with an average size of 20 μm and could be well dispersed in water without obvious deterioration for at least one month. Under visible light, δ-CsPbI_3_@PVP demonstrated an efficient photocatalytic activity, capable of completely degrading a variety of organic dyes, including RhB, MB, and CV, in just 20 min. This high photocatalytic efficiency was attributed to the enhancement of the material’s absorption coefficient in the visible light range arisen from PbI_2_ defects, as well as its low PLQY, allowing for the effective utilization of photogenerated electrons and holes in the photodegradation process. Therefore, δ-CsPbI_3_@PVP microcrystals hold significant potential for the rapid photodegradation of typical organic dyes in the field of photocatalysis. This study provides a new idea for the development of efficient photocatalysts and brings new hope for the research and application in the field of environmental protection and wastewater treatment.

## Figures and Tables

**Table 1:** PLQYs of δ-CsPbI_3_ and δ-CsPbI_3_@PVP using quinine sulfate as a reference.

Sample	Intensity (I)	Abs. (A)	Refractive Index (n)	PLQY (Φ)
Quinine sulfate	12,728.4	0.01	1.33	0.54
δ-CsPbI_3_	813.2	0.202	1.33	0.0017
δ-CsPbI_3_@PVP	2047.4	0.202	1.33	0.0043

**Table 2:** Comparison of photocatalytic activity of perovskite photocatalysts for the degradation of organic pollutants.

Photocatalysts	Contaminants	Test Conditions	Efficiency	Ref.
δ-CsPbI_3_@PVP	RhB in water	visible	99% in 20 min	This work
δ-CsPbI_3_@PVP	MB in water	visible	99% in 20 min	This work
δ-CsPbI_3_@PVP	CV in water	visible	99% in 20 min	This work
CsPbCl_3_ QDs	MO in ethanol	visible	90% in 80 min	[35]
CsPbBr_3_ QDs	MO in ethanol	visible	82% in 80 min	[35]
CsPbBr_3_ CNCs	MB in isopropanol	visible	99% in 60 min	[36]
CsPbBr_3_ crystals	RhB in water	visible	47.3% in 120 min	[44]
CsPb_2_Br_5_ crystals	RhB in water	visible	53.8% in 120 min	[44]
Cs_2_AgBiBr_6_	RhB in ethanol	visible	98% in 120 min	[47]
Cs_2_AgBiBr_6_	Rh110 in ethanol	visible	98% in 280 min	[47]
Cs_2_AgBiBr_6_	MR in ethanol	visible	98% in 420 min	[47]
Cs_2_AgBiBr_6_	MO in ethanol	visible	98% in 300 min	[47]

## Data Availability

Data are contained within the article and Supplementary Materials.

## Supplementary Materials

The following supporting information can be downloaded at: https://www.mdpi.com/article/10.3390/molecules29071670/s1, Figure S1. The length distributions of (a) δ-CsPbI_3_ and (b) δ-CsPbI_3_@PVP microcrystals. Figure S2. The steady-state PL spectra of δ-CsPbI_3_ and δ-CsPbI_3_@PVP powders. The excitation wavelength is 390 nm. Figure S3. Absorption coefficients of TiO_2_, g-C_3_N_4_ and δ-CsPbI_3_@PVP. Figure S4. The visible-light photodegradation of 10 mg/L RhB by δ-CsPbI_3_@PVP microcrystals in water with different PH values.
