# Supplementary material for: PVP Passivated δ-CsPbI3: Vacancy Induced Visible-Light Absorption and Efficient Photocatalysis
Source: Molecules. 2024 Apr 8;29(7):1670. doi: 10.3390/molecules29071670 (PMC11013652; doi:10.3390/molecules29071670)
Supplement: Supplementary file 1 [file molecules-29-01670-s001.zip › molecules-2936003-supplementary.pdf]

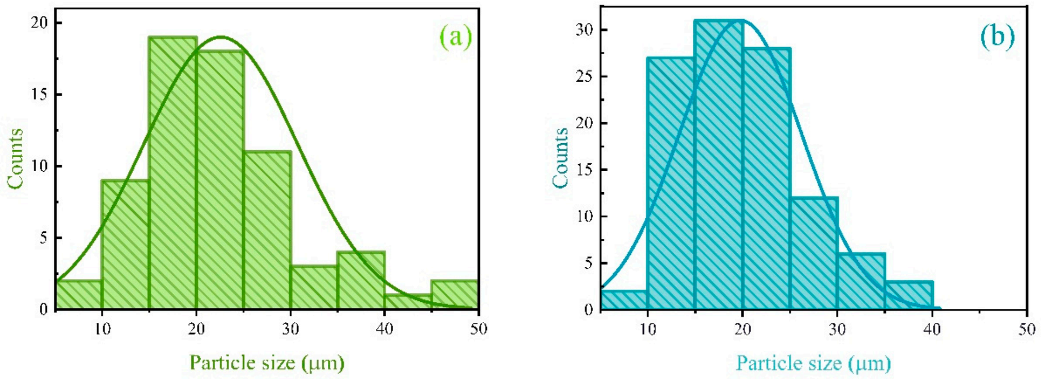

Figure S1. The length distributions of (a) δ-$CsPbI_3$ and (b) δ-$CsPbI_3$@PVP microcrystals.

As shown in Figure S1. the length distributions of δ-$CsPbI_3$ and δ-$CsPbI_3$@PVP microcrystals were measured using Nano Measurer. The average length of δ-$CsPbI_3$ microcrystals is 22.5 microns (Figure S1a). The average length of δ-$CsPbI_3$@PVP microcrystals is 20 microns (Figure S1b).

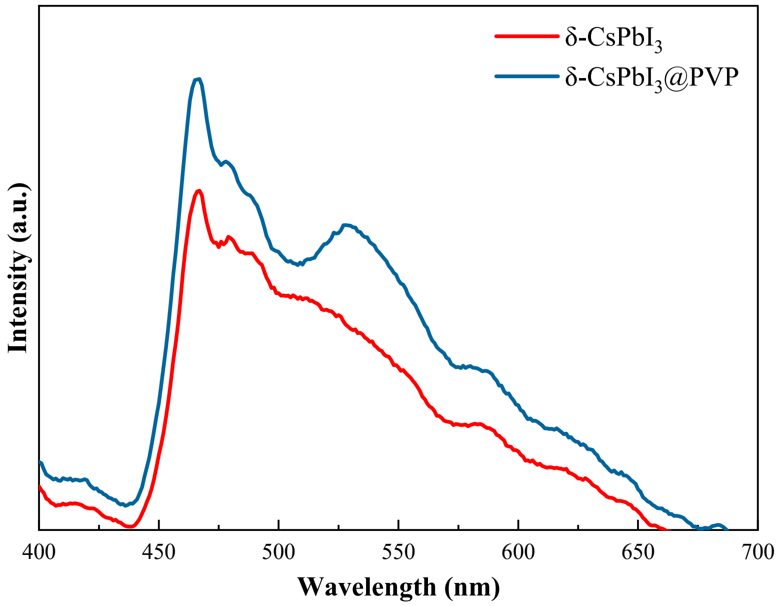

Figure S2. The steady-state PL spectra of δ-$CsPbI_3$ and δ-$CsPbI_3$@PVP powders. The excitation wavelength is 390 nm.

As shown in Figure S2. under the 390 nm excitation condition, the steady-state emission spectra (PL) of δ-$CsPbI_3$ and δ-$CsPbI_3$@PVP powders are both broad peaks from 437—660 nm, with the peak near 466 nm coming from exciton emission, while δ-$CsPbI_3$@PVP powders have a new peak near 530 nm, and the new peak may be from the self-trapped state.

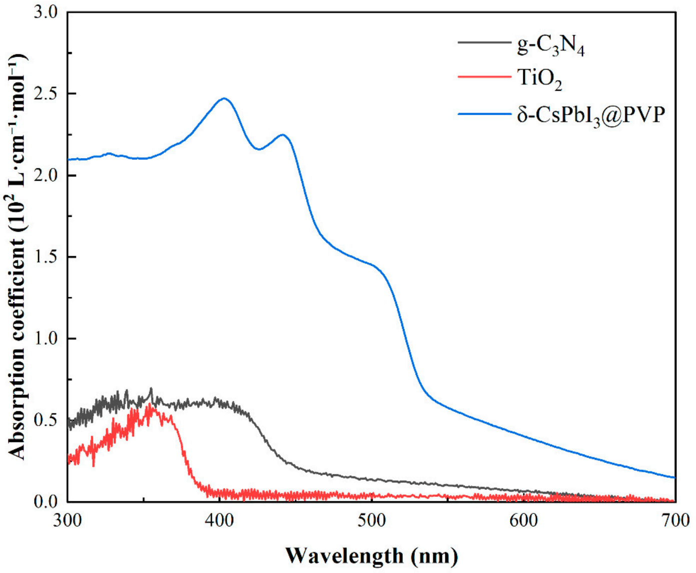

Figure S3. Absorption coefficients of TiO_2, g-C_3N_4 and δ-$CsPbI_3$@PVP.

Figure S3. The molar absorption coefficients of TiO_2, g-C_3N_4 and δ-$CsPbI_3$@PVP were obtained by measuring their absorption spectra and calculated according to formula (2). Among them, the aqueous concentrations of TiO_2, g-C_3N_4 and δ-$CsPbI_3$@PVP are 9.39×10^{-3} mol/L , 1.63×10^{-3} mol/L and 4.16×10^{-3} mol/L, respectively. The results show that the absorption coefficients of δ-$CsPbI_3$@PVP are 2—5 times larger than those of TiO_2, g-C_3N_4 within the visible range of 400—530 nm.

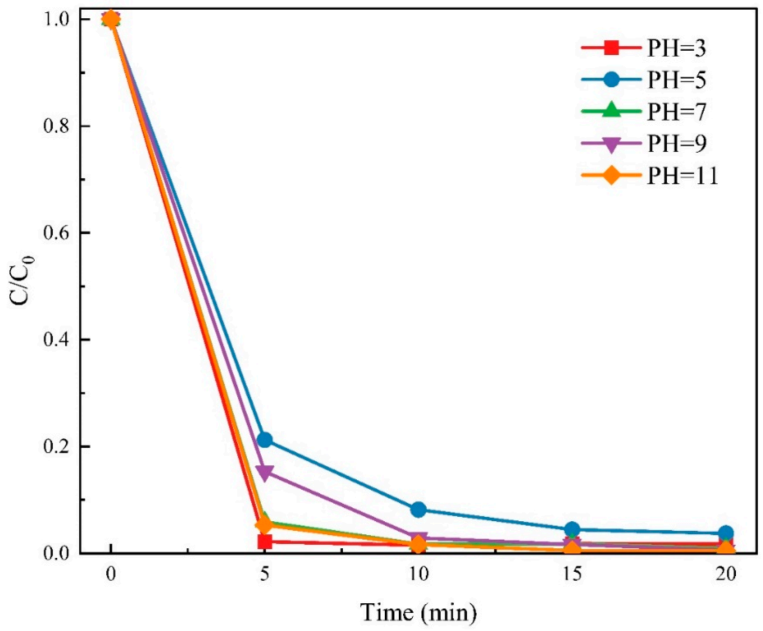

Figure S4. The visible-light photodegradation of 10 mg/L RhB by δ-$CsPbI_3$@PVP microcrystals in water with different PH values.

Different from other photocatalytic organic dye degradation experiments, 1mol/L NaOH and 1mol/L HCl were used to adjust the PH of the solution to 3, 5, 7, 9, 11. The experimental results are shown in Figure S4. The change of PH does not affect the degradation of 99% RhB by δ-$CsPbI_3$@PVP within 20 min.
